# Challenges for maintaining surgical care practices in the COVID-19 pandemic: an integrative review*

**DOI:** 10.17533/udea.iee.v40n1e16

**Published:** 2022-03-30

**Authors:** José Erivelton de Souza Maciel Ferreira, Tahissa Frota Cavalcante, Raphaella Castro Jansen, Daniel Freitas Oliveira Damasceno, Lídia Rocha de Oliveira, Maria Jocelane Nascimento da Silva, Ana Paula Rodrigues

**Affiliations:** 1 Nurse, Master’s student. Email: eriveltonsmf@gmail.com. Universidade da Integração Internacional da Lusofonia Afro-Brasileira, Brazil. Universidade da Integração Internacional da Lusofonia Afro-Brasileira Universidade da Integração Internacional da Lusofonia Afro-Brasileira Brazil eriveltonsmf@gmail.com; 2 Nurse, Ph.D. Adjunct Professor. Email: tahissa@unilab.edu.br Universidade da Integração Internacional da Lusofonia Afro-Brasileira, Brazil. Universidade da Integração Internacional da Lusofonia Afro-Brasileira Universidade da Integração Internacional da Lusofonia Afro-Brasileira Brazil tahissa@unilab.edu.br; 3 Nurse, lato sensu graduate student. Brazil. Email: raphaella.jansen@gmail.com Universidade da Integração Internacional da Lusofonia Afro-Brasileira, Brazil. Universidade da Integração Internacional da Lusofonia Afro-Brasileira Universidade da Integração Internacional da Lusofonia Afro-Brasileira Brazil raphaella.jansen@gmail.com; 4 Nurse, Master’s student. Email: danielfreitas17@yahoo.com.br Universidade da Integração Internacional da Lusofonia Afro-Brasileira, Brazil. Universidade da Integração Internacional da Lusofonia Afro-Brasileira Universidade da Integração Internacional da Lusofonia Afro-Brasileira Brazil danielfreitas17@yahoo.com.br; 5 Nurse, Master’s student. Email: lidiarocha2021@gmail.com Universidade da Integração Internacional da Lusofonia Afro-Brasileira, Brazil. Universidade da Integração Internacional da Lusofonia Afro-Brasileira Universidade da Integração Internacional da Lusofonia Afro-Brasileira Brazil lidiarocha2021@gmail.com; 6 Nurse, Master’s student. Email: jocelane.nascimento.silva@gmail.com Universidade da Integração Internacional da Lusofonia Afro-Brasileira, Brazil. Universidade da Integração Internacional da Lusofonia Afro-Brasileira Universidade da Integração Internacional da Lusofonia Afro-Brasileira Brazil jocelane.nascimento.silva@gmail.com; 7 Physical education, Ph.D. Universidad de la Empresa, Uruguay. Email: anapaula_apr@hotmail.com.br Universidad de la Empresa Universidad de la Empresa Uruguay anapaula_apr@hotmail.com.br

**Keywords:** surgicenters, coronavirus infections, delivery of healthcare, health management, nursing., centros quirúrgicos, infecciones por coronavirus, atención a la salud, gestión en salud, enfermería., Centros Cirúrgicos, Infecções por Coronavírus, Assistência à Saúde, Gestão em Saúde, Enfermagem.

## Abstract

**Objective.:**

To present the knowledge produced on challenges of health services for maintaining surgical care practices in times of the COVID-19 pandemic.

**Methods.:**

This is an integrative literature review, performed with descriptors ‘Operating rooms’ and ‘Coronavirus Infections’ in the MEDLINE/PubMed Central, IBECS, LILACS, BDENF, *Coleta SUS*, BIGG, BINACIS, SciELO, PubMed, Science Direct, and Cochrane Library databases.

**Results.:**

Of the 405 studies analyzed, 27 met the inclusion criteria. The main challenges for surgical services during the pandemic were: (i) rearrangement of general practice in surgical units; (ii) administration and management of resources and elective surgeries; (iii) follow-up and control of preoperative patients to medium term; (iv) maintenance of patients’ and health professionals’ autonomy and mental health in this context; and (v) teaching health residents in the operating room.

**Conclusion.:**

For surgical care services, the challenges caused by managing the high demand of patients in need of care resulted in the transfer of own resources to other units and the consequent hiring of professionals to meet the demand for these services due to the damming of postponed elective surgeries. This knowledge will allow us to propose strategies in decision making in this scenario, considering the new waves that may arise from this disease.

## Resumen

**Objetivo.:**

Presentar el conocimiento producido sobre los desafíos de los servicios de salud para el mantenimiento de las prácticas de atención quirúrgica en tiempos de pandemia por COVID-19.

**Métodos.:**

Revisión integradora de la literatura, realizada con los descriptores ‘Centros quirúrgicos’ e ‘Infecciones por Coronavírus’ en las bases de datos Medline/PubMed Central, IBECS, LILACS, BDENF, Collects SUS, BIGG, BINACIS, SCIELO, PUBMED, SCIENCE DIRECT y Cochrane Library.

**Resultados.:**

De 405 estudios analizados 27 cumplieron los criterios de inclusión. Los principales desafíos para los servicios de cirugía durante la pandemia fueron: (i) la reorganización de la práctica del cuidado general en las unidades quirúrgicas, (ii) la gestión y administración de recursos y de las cirugías electivas, (iii) el seguimiento y control de los pacientes preoperatorios a medio plazo, (iv) el mantener la autonomía y la salud mental del paciente y de los profesionales sanitarios en este contexto, y (v) la enseñanza de los residentes sanitarios en el quirófano.

**Conclusión.:**

Para los servicios de atención quirúrgica los retos ocasionados por el manejo de la alta demanda de pacientes con necesidad del cuidado tuvo como consecuencia la transferencia de recursos propios a otras unidades y la contratación posterior de profesionales para suplir la demanda de estos servicios ante el represamiento de cirugías electivas pospuestas. Este conocimiento permitirá plantear estrategias en la toma de decisiones frente a este escenario, considerando las nuevas olas que se pueden presentar de esta enfermedad.

## Resumo

**Objetivo.:**

Apresentar o conhecimento produzido sobre os desafios dos serviços de saúde para a manutenção das práticas de cuidados cirúrgicos em tempos de pandemia por COVID-19.

**Métodos.:**

Revisão integrativa da literatura, realizada com os descritores ‘Centros Cirúrgicos’ e ‘Infecções por Coronavírus’ nas bases de dados Medline/PubMed Central, IBECS, LILACS, BDENF, Coleta SUS, BIGG, BINACIS, SCIELO, PUBMED, SCIENCE DIRECT e Cochrane Library.

**Resultados.:**

Dos 405 estudos analisados, 27 preencheram os critérios de inclusão. Os principais desafios para os serviços cirúrgicos durante a pandemia foram: (i) a reorganização da clínica geral nas unidades cirúrgicas, (ii) a gestão e administração de recursos e cirurgias eletivas, (iii) o acompanhamento e controle de pacientes pré-operatórios a médio prazo, (iv) manutenção da autonomia e saúde mental do paciente e dos profissionais de saúde nesse contexto, e (v) ensino dos residentes de saúde no centro cirúrgico.

**Conclusão.:**

Para os serviços de atendimento cirúrgico, os desafios causados pelo gerenciamento da alta demanda de pacientes com necessidade de atendimento resultaram no repasse de recursos próprios para outras unidades e consequente contratação de profissionais para atender a demanda desses serviços devido ao represamento de cirurgias eletivas adiadas. Este conhecimento permitirá propor estratégias na tomada de decisão diante desse cenário, considerando as novas ondas que podem surgir dessa doença.

## Introduction

This research aims to investigate the challenges that surgical health services have faced in the face of the COVID-19 pandemic in order to preserve safe and effective multidisciplinary healthcare. Infection with SARS-CoV-2, the etiological agent that causes the disease COVID-19, affects an exponentially growing number of individuals daily.(1) Due to its potential for transmission, contagion and lethality, a high number of people were infected worldwide, becoming responsible for the collapse of public and private services in the health systems of many countries. Due to this set of factors, the World Health Organization (WHO) declared the COVID-19 pandemic.(2) The clinical picture of affected cases varies from asymptomatic to mild, moderate and severe symptoms, and may progress to critical respiratory failure and even multiple organ failure.(3,4)

The COVID-19 pandemic has impacted all health sectors, with the need to issue some recommendations and guidelines for surgical practice and patient management during this new phase. WHO released the guideline for triage of surgical patients, prioritizing emergency surgeries and postponing elective procedures until the pandemic stabilizes.(5) As a result, due to the lack of effective treatments and even the immunizing vaccine at the time, many hospitals and surgical clinics had to restructure and rearrange their processes and care.(6) Many countries have designated hospitals and other physical health services for the management of patients infected with the disease.(7) Moreover, health authorities have mobilized the adoption of measures that meet the arrangement and assistance of these services, especially those that provide intensive care, since they are the most overloaded in the current context.(8) In view of this, challenges arise for the safe operation of these health services and with them the need to devise strategies for the arrangement and/or rearrangement of these spaces, including operating rooms or outpatient clinics.(9)

Studies show that the volume of surgical emergencies has dropped dramatically. However, in response to the pandemic, several hospitals around the world needed to prepare to meet a high demand from COVID-19 patients, many of whom required specialized care in ICUs. The most widespread recommendation by scholars was that surgical departments prepare for changes in the acute surgical needs of the population they assist, which could allow for a better allocation of limited resources.(10,11) Given this context, in order to follow institutional technical standards for disease prevention and control during surgical care practices throughout the perioperative period, it is believed that these services continue to overcome managerial, cultural, philosophical and structural challenges based on effective and safe strategies to maintain their proper functioning. Listing these challenges is important so that professionals from the surgical team understand the motivations that forced changes in their work routine and so that they can reflect on the different strategies to overcome the challenges also arising from these changes.

The innovative character of this research is supported by the fact that the proposed theme has gained international repercussion. Therefore, considering the relevance of the explained content, presenting the knowledge that was produced in those times is important for surgical teams to have this knowledge to understand and transform their care and management practice in the current pandemic context. Thus, this study aimed to present the knowledge produced about the challenges of health services for maintenance of surgical care practices in times of the COVID-19 pandemic.

## Methods

This is an integrative literature review, characterized as a research method that makes it possible to gather, analyze and synthesize available research on certain topics in a systematic way. To prepare this integrative review, the following steps were followed: 1) theme identification and research guiding question construction; 2) problem and study objective identification; 3) literature search; 4) data collection; 5) critical analysis of results; and 6) presentation of synthesis.(12) The topic of interest was the arrangement of health services with regard to surgical care practices in times of the COVID-19 pandemic. The PICo strategy (Problem - Interest - Context) was used to formulate the guiding question as follows: What have been the challenges of health services for the arrangement of surgical care practices during the COVID-19 pandemic?

The data collection stage was carried out from consultations in the PubMed portal, the regional portal of the Virtual Health Library (VHL), in the academic search engine Science Direct, in the Cochrane database and in the Scientific Electronic Library Online (SciELO). The databases accessed from VHL were BDENF, LILACS, MEDLINE, IBECS, *Coleciona SUS*, BIGG, and BINACIS. For the databases accessed from the VHL, the Brazilian Health Science Descriptors (DeCS) were adopted, which were crossed as follows: “*Centros Cirúrgicos*” AND “*Infecções por Coronavírus*”. For other consultations, controlled descriptors from the Medical Subject Headings (MeSH) of U.S. National Library of Medicine and the following crosses were performed: “Surgicenters” AND “Coronavirus Infections”. In order to identify only articles that presented both terms in the same study, it was necessary to use the crossing between the descriptors with the Boolean operator “AND”.

Scientific articles published and available electronically in full and that answered the guiding question of this research were included. The access to studies that were not available in full in the databases took place from the Coordination for the Improvement of Higher Education Personnel platform (CAPES - *Coordenação de Aperfeiçoamento de Pessoal de Nível Superior*). Articles that were repeated in the databases were excluded. The bibliographic search stage took place between February and April 2021 and the databases were accessed through a computer from a university linked to the world wide web, which were exhausted in a single day with recording of the search page. Study search and selection was paired.

Below, in Table 1, are the results of bibliographic search in the databases, where the proper distribution of articles found and selected is described.

**Table 1:** Quantitative distribution of found and selected articles

	Database	Articles found	Articles deleted after text skimming	Articles selected for full reading	Articles that answer the guiding question
Virtual Health Library	MEDLINE	352 (86.91%)	284 (70.12%)	68 (16.79%)	24 (5.92%)
Virtual Health Library	IBECS	20 (4.93%)	15 (3.7%)	5 (1.23%)	1 (0.24%)
Virtual Health Library	LILACS	15 (3.7%)	9 (2.22%)	6 (1.48%)	0
Virtual Health Library	BDENF	4 (0.98%)	2 (0.49%)	2 (0.49%)	1 (0.24%)
Virtual Health Library	*Coleciona SUS*	2 (0.49%)	1 (0.24%)	1 (0.24%)	0
Virtual Health Library	*BIGG - Guias* GRADE	1 (0.24%)	1 (0.24%)	0	0
Virtual Health Library	BINACIS	1 (0.24%)	0	1 (0.24%)	0
Total		395 (97.53%)	312 (77.03%)	83 (20.49%)	26 (6.41%)
International	SCIELO	1 (0.24%)	0	1 (0.24%)	0
International	PUBMED	6 (1.48%)	2 (0.49%)	4 (0.98%)	1 (0.24%)
International	SCIENCE DIRECT	3 (0.74%)	2 (0.49%)	1 (0.24%)	0
International	COCHRANE	0	0	0	0
Total		10 (2.46%)	4 (0.98%)	6 (1.48%)	1 (0.24%)
Overall total		405 (100%)	318 (78.51%)	87 (21.48%)	27 (6.66%)

It is observed that a total of 405 articles were found. All were read from a dynamic, and text skimming was carried out by the appreciation of titles and abstracts, selecting those that had an interface with the subject of study for a full reading. Of these, 318 (78.51%) were excluded because they were duplicate manuscripts, were not articles or did not respond to the guiding question of this review. A total of 87 (21.48%) articles related to the subject were exhaustively analyzed; of these, 27 (6.66%) answered the guiding question. The identification, screening and inclusion processes are outlined in Flowchart 1.

**Flowchart 1:**
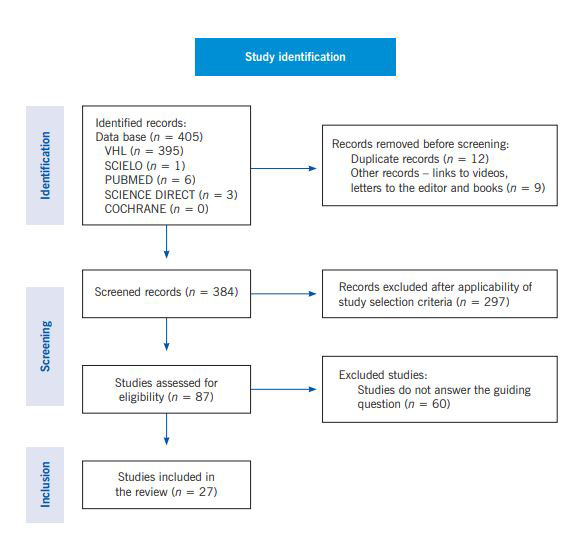
Flowchart based on the PRISMA**(**13) model with the results of article selection

A validated data collection form was used to collect data from each article in the final sample and this instrument allowed the acquisition of information about article identification, type of publication, methodological characteristics of the study and level of evidence.(14) The same instrument was used in other integrative literature reviews.(15,16) Regarding the level of evidence, the studies were classified according to the Melnyk and Fineout-Overholt design.(17) This design works with the following levels of evidence: 1 - evidence from a systematic review, meta-analysis or clinical guidelines from systematic reviews of randomized and controlled clinical trials; 2 - evidence from at least one randomized controlled clinical trial; 3 - evidence from well-designed clinical trials without randomization;4 - evidence from a well-designed cohort and case-control study; 5 - evidence presented from a systematic review, from descriptive and qualitative studies; 6 - evidence from a single descriptive or qualitative study; 7 - evidence derived from opinion articles. Furthermore, the results were analyzed based on content and thematic analysis,(18) which were arranged into a single category, in the form of a table entitled as challenges for health services to rearrange surgical units.

## Results

In Table 2, it is possible to obtain the main information of selected studies: identification, database, journal, year of publication, level of evidence and title corresponding to the information recorded.

**Table 2:** Selected national and international scientific production

Database	Journal and Year of publication	Level of evidence	Article title	Reference
IBECS	*Revista Española de Cirugía Ortopédica y Traumatológica* (2021)	Level 6	*Resultado de la implantación de consultas telemáticas en cirugía ortopédica y traumatología durante la pandemia COVID-19*	19
BDENF	*Sociedade Brasileira de Enfermagem de Operating room* (2020)	Level 7	Training and qualification of nursing professionals in the operating room to care for patients infected with SARS-CoV-2 in external areas	20
MEDLINE	The Annals of The Royal College of Surgeons of England (2021)	Level 4	St Andrew’s COVID-19 surgery safety study: hand trauma.	21
MEDLINE	PloS one (2020)	Level 4	Surgery for non-Covid-19 patients during the pandemic.	22
MEDLINE	BMC Medicine (2020)	Level 5	Adapting hospital capacity to meet changing demands during the COVID-19 pandemic.	23
MEDLINE	Acta neurochirurgica (2020)	Level 4	Intensive care of traumatic brain injury and aneurysmal subarachnoid hemorrhage in Helsinki during the Covid-19 pandemic	24
MEDLINE	Journal of Medical Ethics (2020)	Level 7	Maternal request caesareans and COVID-19: the virus does not diminish the importance of choice in childbirth	25
MEDLINE	*Revista Española de anestesiologia y reanimación* (2020)	Level 6	Experience of a pediatric monographic hospital and strategies adopted for perioperative care during the SARS-CoV-2 epidemic and the rearrangement of urgent pediatric care in the Community of Madrid.	26
MEDLINE	European Journal of Cardio-Thoracic Surgery (2020)	Level 4	Clinical features and outcomes of thoracic surgery patients during the COVID-19 pandemic.	27
MEDLINE	*Actas Urológicas Españolas* (2020)	Level 6	Design of an assistance protocol for the restart of scheduled urologic surgery in a COVID-19 epidemic period.	28
MEDLINE	Anaesthesya (2020)	Level 4	The safety of paediatric surgery between COVID-19 surges: an observational study.	29
MEDLINE	Canadian Medical Association Journal (2020)	Level 4	Clearing the surgical backlog caused by COVID-19 in Ontario: a time series modelling study.	30
MEDLINE	Langenbeck’s archives of surgery (2020)	Level 4	Transforming a surgical department during the outbreak of new coronavirus pandemic. Clinical implications	31
MEDLINE	Plastic and Reconstructive Surgery (2020)	Level 6	The Early Effects of COVID-19 on Plastic Surgery Residency Training: The University of Washington Experience	32
MEDLINE	Anaesthesia Critical Care & Pain Medicine (2020)	Level 7	How to resume elective surgery in light of COVID-19 post-pandemic propofol shortage: The common concern of anaesthesists and surgeons.	33
MEDLINE	World neurosurgery (2020)	Level 6	Adapting Neurosurgery Practice During the COVID-19 Pandemic in the Indian Subcontinent.	34
MEDLINE	Annals of surgery (2020)	Level 7	Operationalizing the Operating Room: Ensuring Appropriate Surgical Care in the Era of COVID-19.	35
MEDLINE	Pediatric surgery international (2020)	Level 6	Challenges to delivering pediatric surgery services in the midst of COVID 19 crisis: experience from a tertiary care hospital of Pakistan.	36
MEDLINE	International Journal of Surgery (2020)	Level 6	Surgical activity during the Covid-19 pandemic: Results for 112 patients in a French tertiary care center, a quality improvement study.	37
MEDLINE	Pediatric Surgery International (2020)	Level 6	Assess, adapt and act: a paediatric surgery division’s initial approach in a rapidly evolving pandemic.	38
MEDLINE	International Journal of Surgery (2020)	Level 6	Perspectives on how to navigate cancer surgery in the breast, head and neck, skin, and soft tissue tumor in limited-resource countries during COVID-19 pandemic.	39
MEDLINE	Updates in surgery (2020)	Level 6	Continuing our work: transplant surgery and surgical oncology in a tertiary referral COVID-19 center.	40
MEDLINE	Clinics (2020)	Level 6	Transforming operating rooms into intensive care units and the versatility of the physician anesthesiologist during the COVID-19 crisis.	41
MEDLINE	New England Journal of Medicine (2020)	Level 7	Surgery Scheduling in a Crisis.	42
MEDLINE	Anesthesia and analgesia (2020)	Level 7	COVID-19: Role of Ambulatory Surgery Facilities in This Global Pandemic.	43
MEDLINE	*Cirugía Española* (2020)	Level 4	SARS-CoV-2 pandemic on the activity and professionals of a General Surgery and Digestive Surgery Service in a tertiary hospital.	44
PUBMED	Journal of Thoracic Oncology (2020)	Level 6	Coronavirus Disease 2019 in the Perioperative Period of Lung Resection: A Brief Report From a Single Thoracic Surgery Department in Wuhan, People’s Republic of China.	45

It can be noted that most of the selected studies come from databases accessed through the VHL (n = 26; 96.29%), mainly MEDLINE (n=24; 88.88%). Most were published in 2020 (n=25; 92.59%) and the others were published in 2021 (n=2; 3.3%). The journals that published the most on the researched topic and that answered the study question were those in the medical field (n=25; 92.59%). The studies are available in English, Spanish or Portuguese, most of them in English (n=26; 96.29%). As for the level of evidence of the selected articles, it was observed that 12 (44.44%) studies bring evidence of level 6, that is, derived from descriptive or qualitative studies. Below, Table 3 presents the challenges that healthcare services have faced and have faced in maintaining surgical care practices during the COVID-19 pandemic. The synthesis of results, in this table, was arranged into six categories, related to the following aspects: rearrangement of general care practices in surgical health units; challenges related to administration and management of the surgical sector; follow-up and control of mediate preoperative patients; patients’ and health professionals’ autonomy and mental health in this scenario; teaching and learning of multidisciplinary health residents in the operating room; and financial maintenance of public and private surgical health services.

**Table 3:** Challenges for health services to rearrange surgical care practices during the COVID-19 pandemic

Challenges of health services to rearrange the surgical units	***n* (%)**	References
*Challenges in rearranging general care practices in surgical health facilities*		
Execution of resolutive service in a single consultation	1 (3.7)	33
Increased attributions to the perioperative service nurse	1 (3.7)	20
Decision making to ensure the safety of patients and healthcare professionals during this challenging period	1 (3.7)	21
Readjustment of routine surgical practices	1 (3.7)	27
Hiring doctors and nurses to meet the demands of the operating room and other units	5 (18.51)	22, 38, 41, 42, 44
Overcoming the negative impacts on the care system for surgical patients with cancer or patients in need of transplants	3 (11.1)	39, 44, 45
*Challenges related to administration and management of resources,* *beds and elective surgeries in the surgical sector*		
Managing the high demand of patients requiring perioperative care	3 (11.1)	2, 33, 35
Transfer of material and human resources from the operating room to emergency units and ICUs	7 (25.92)	22, 24, 26, 31, 39-41
Management of the accumulation of postponed elective surgeries and the impacts of this waiting on patients’ lives	6 (22.22)	22, 30, 31, 36, 37, 44
Supply and guarantee of surgical beds for all users	1 (3.7)	23
*Challenges in follow-up and control of mediate preoperative patients*		
Decision on the return of scheduled surgical activities	2 (7.4)	28, 29
Follow-up and control of clinical conditions of patients who have had their surgeries postponed	4 (14.81)	31, 39, 44, 45
*Challenges in maintaining patients’ and health professionals’ autonomy* *and mental health in this scenario*		
Maintaining patients’ autonomy, including women, as to the choice of their type of childbirth (caesarean or not)	1 (3.7)	25
Maintenance of mental health and overcoming the shaken emotional aspects of surgical teams and patients	2 (7.4)	33, 34
Dismantling the waves of false information that surgical patients had access to about the COVID-19 disease	2 (7.4)	33, 34
*Challenges in teaching multidisciplinary operating room health residents during the pandemic*		
Maintaining continued and supervised education of residents	1 (3.7)	32
*Challenges in the financial maintenance of public and private surgical* *health services*		
Control of impacts that negatively affect private outpatient health services that offer elective procedures: loss of revenue and personnel	1 (3.7)	43

All selected articles brought at least one piece of information that was categorized as one of the challenges for health services that provide surgical care, regardless of whether it is public or private, to the detriment of the COVID-19 pandemic. The most cited challenges were management of the high demand of patients in need of perioperative care (11.1%), accumulation of elective surgeries postponed due to the COVID-19 pandemic (22.2%), transfer of resources from surgical centers to other units (25.9%) and hiring of professionals to meet the demands of these health services that provide surgical care (18.5%). Follow-up and controlling the clinical conditions of patients who had their surgeries postponed was also an important challenge emphasized (14.8%).

## Discussion

### Rearrangement of general care practices in surgical health units

As a result of the pandemic caused by the new SARS-CoV-2, health services that provide surgical care needed to reorganize their care practices. This required technical, scientific and management skills and knowledge on the part of those who run hospital and/or outpatient surgical units. A study(19) pointed out that, in the current pandemic context, surgical services needed to perform their care in a resolute way in a single consultation, especially with regard to medical and nursing surgical consultations. In this way, patients would be less exposed to the virus, as they would leave the house only once to solve their health demand and would be less exposed to the virus in the hospital or outpatient environment, reducing their chances of being infected by the new coronavirus.

It is known that surgical consultations are unpredictable, therefore, identifying patients’ problems and solving them immediately will not always be possible, and, in these cases, there is a special interdependence between the client and the surgical team. Due to this fact, patients often end up traveling between many health services to have their complaint resolved as quickly as possible.(46) Thus, if a patient attends a preoperative consultation without presenting the requested or legible exams or with the absence of some important document, for example, it is a challenge for qualified professionals to continue with this service, even though they understand the current public health chaos. Some institutions were unable to employ resources, such as telemedicine, to solve this problem, while others were successful.(47)

More than ever before, this new era requires healthcare and management professionals to become increasingly skilled in their decision-making to ensure the safety of clients who seek help and professionals who risk their health to stay working.(21) Certainly, this is one of the main challenges that health services around the world face, including surgical ones. In the same proportion that the client lacks safe, error-free and damage-free care, professionals who offer this care must be well directed, in terms of their conduct, to remain free from legal proceedings, violence and health problems. More has been demanded of health professionals in these times, so keeping them productive amid pandemic tension and the increase in activities assigned to them is a challenge. In this scenario, surgical team professionals whose clinical-surgical attributions were expanded in order to meet the health service personnel’s needs stand out.(20)

The current working conditions of health professionals, especially nurses, in many countries around the world, are marked by overload of activities, low remuneration and insufficient availability of personal protective equipment.(48) These factors are certainly the main responsible for the exhaustion, contamination, illness and death of these professionals who work courageously on the front line, whether in the wards, in the surgical centers or in ICUs. Many professionals at the beginning of the pandemic were removed because they had risk factors for COVID-19 and others continue to be contaminated, falling ill and/or dying, further reducing the number of professionals to work on the front line of the disease. This scenario has increasingly demanded that health units hire doctors and nurses to meet the demands of the operating room and other units.(22,38,41,42,44)

It is important to emphasize that, since graduation, there has always been a discussion about the number of insufficient professionals in health services to provide safe and resolute care. With the pandemic, it could be inferred that a worsening of this framework of insufficient professionals is primarily responsible for culminating in work overload for those who remain working, given the high demand of patients for urgent care.

### Challenges related to administration and management of the surgical sector

The difficulty faced by health units to manage the high demand of patients who require urgent perioperative care was one of the challenges highlighted, and this required important resignifications in this sector, even if temporary. Scholars(20,33,35) reveal that the high demand for patients and care, in public or private institutions, required the support of perioperative nursing to provide intensive care to patients in need of critical care in the anesthetic recovery rooms, because they ended up admitting critical postoperative patients indicative of ICU admission. Managing a surgical sector in these conditions was extremely delicate.

One of the greatest concerns of managers of these health services was regarding the control and availability of beds for intensive care for victims of COVID-19.(11) This led the management teams of these institutions, at the global level, to envision ways to transfer material and human resources from the operating room to the first aid units and ICUs.(22,24,26,31,39,40,41) Concomitantly, these managers were also concerned with providing enough emergency surgical beds to meet the demand of users who needed it.(23) With material and human resources already limited in all sectors, it was certainly challenging for managers to decide the best strategies for relocating personnel and materials at this unit to those whose service demand was higher. It is also possible to reflect that managing surgical units with fewer professionals and materials has also become challenging, even more so with the condition of keeping emergency surgical beds available for patients with COVID-19.

In the waves of contagion of the disease, elective surgeries had to be postponed. As a result, the number of elective surgeries generated an increasing volume of backlogs. Managing this accumulation and the impacts of this waiting on the lives of patients worried health services.(22,30,31,36,37,44) It became necessary, therefore, to devise ways to overcome the negative impacts on the system of care for elective surgical patients, especially those with important chronic conditions such as cancer or who need transplants.(39,44,45) Studies indicate that patients with long-deferred elective surgical indications can evolve to a poor prognosis and, at times, can be fatal. Thus, assessing the feasibility of performing elective surgeries during the COVID-19 pandemic has been extremely necessary, especially during the waves of contagion. Certain clinical conditions and diseases, especially those that present stages of evolution, can progress to a degree that manifests organic responses that cause suffering to patients and irreversible damage to patients.(49) It is also recommended that cancer patients or those in need of transplants should not have their surgeries postponed.(49,50)

Due to the cases of patients who presented a bad evolution of their clinical condition because their surgeries were postponed, the need to follow up and control the clinical conditions of all patients who had their surgeries postponed because they were classified as elective.(31,39,44,45) Administration and management of all these nuances was of utmost importance during the peaks of the waves of contagion and hospitalization of patients by COVID-19.

### Follow-up and control of mediate preoperative patients

The researchers(51) revealed that following up patients with surgical indication has become a challenge because many patients, during peaks of contagion, do not attend their control and routine appointments and, as a result, follow-up visits are seriously interrupted. In this case, one should consider the fear that these patients have of being contaminated when seeking health services during this period, even recognizing the worsening of their health condition. Thus, considering the concern of health professionals with these patients and the negative clinical outcome of these subjects, reflections began to drive the idea that scheduled surgical activities should be restarted.(28,29)

Deciding to resume elective surgical activities in the pandemic scenario, especially amidst the waves of contagion, requires constant observation about the readjustment of institutional routine surgical practices. This is necessary in order to ensure the safety of assisted patients and healthcare professionals. This challenge requires an important dedication from all those involved in the assistance and from those who are in charge of developing protocols and guidelines for institutional care.(27) Guidance on the return of essential and non-essential surgeries during the COVID-19 pandemic continues to be carried out, especially after the peaks of contamination by the new SARS-CoV-2. In some countries, the recommendation, before restarting elective surgical programming after a wave of contamination of the population by COVID-19, is to carry out a survey of the occupancy rate of ICU beds, analyze the moment of resumption, considering the incidence rate of COVID-19, flow of care and protocols registered in regional medical councils, in order to always maintain autonomy of patients to the best of their ability.(52,53)

### Patients’ and health professionals’ autonomy and mental health in this scenario

The maintenance of patients’ autonomy in the context of healthcare in these times of pandemic was widely discussed, including that of women in choosing their type of childbirth.(25) Therefore, the elaboration of care protocols to be implemented in childbirth, puerperium and abortion during the current pandemic in agreement with the restrictions imposed by institutional guidelines on surgical activities during peaks of contagion by this disease has been considered important, respecting the autonomy of women throughout the puerperium or abortion process.(54,55) Although the current situation seems to haunt health professionals, it is important to evoke the importance that caring holistically involves humanization. The autonomy of no patient and health professional should be questioned or disrespected.

Another important challenge to be discussed is mental health and overcoming the shaken emotional aspects of patients and professionals who make up the surgical team.(33,34) Those who care for the sick may become mentally ill; this emotional exhaustion can favor the loss of team personnel and the increase of professionals with mental disorders acquired as a result of the COVID-19 pandemic, with special emphasis on those who make up the nursing team.(56) It is important that the institutions themselves provide psychological support services for these professionals, so that they follow them up until discharge and return to activities.

Surgical patients are not exempt from being mentally affected, especially in the era of fake news, a challenging period for health professionals to be able to demystify the waves of false information that spread about the COVID-19 disease.(33,34) Unconventional issues such as loss of economic pay, mental health concerns, the impact of social media, and the wave of surveys and webinars are the main factors affecting the mental health of these patients. Violence and threats to health professionals are also due to access to false information about scientifically proven treatment and conduct.(34)

In professional practice, patients are commonly seen emotionally hesitant, insecure and fluctuating about their thoughts and attitudes as a result of their surgical condition. In addition to these factors that affect the mental health of these patients, it is important to point out that professionals from the surgical team and their residents should pay attention to this situation of overlapping risk factors for mental illness. This is important so that these clients do not give up on their treatment and/or do not fail to adhere to the healthcare prescribed by the multidisciplinary team throughout or at some specific time during the perioperative period. Studies show that psychology residents have contributed significantly to this situation.(57)

### Teaching and learning of multidisciplinary health residents in the operating room

Health residents’ professional performance in surgical units is essential for holistic healthcare and for controlling the demand of patients who need clinical-surgical care. However, the education and arrangement of these professionals in these times of pandemic was seriously compromised at the peak of the disease.(32,58) These resident professionals from different professions, whether in nursing, medicine, psychology, physiotherapy, among others, have been continuously acting in the face of COVID-19, even with the limitations imposed by the pandemic.(59-61) However, due to professional inexperience, these resident professionals and trainees may be more exposed to the disease, and that is why the responsible institutions and their supervisors must continuously offer them training and qualification courses to face the pandemic over time in these services, even if they are vaccinated. It is also necessary to encourage more reflections on teaching strategies in the multidisciplinary residency that should be adopted, with a focus on overcoming the challenges imposed in the teaching and learning of these agents.

In this scenario, the presence of tutors responsible for these subjects is essential so that they can supervise the professional practice of these health residents with excellence. Surgical patients need quality care that prevents and minimizes the risks involved during surgery. Patients with COVID-19 or with COVID-19 sequels during infection or post-infection, with or without sequels, need multidisciplinary residents and qualified institutional staff.

### Financial maintenance of public and private surgical health services

Finally, it should be noted that the impacts of this pandemic have not only affected the community and public health institutions, but also private outpatient services that offer elective procedures. The main challenges focused on overcoming the loss of revenue and personnel for some of these units, especially for public services.(43) These health units have suffered considerable economic impacts, being more significant for public services, since they need a minimum income to pay for their health personnel employed and materials. Although it was seen that many health institutions hired more professionals, many others had to be fired due to the suspension of elective procedures, which led to a decrease in the income of the institutions for this sector. Revenues that were previously destined for public health clinics, for instance, needed to be reallocated to the structuring and operationalization of field hospitals.(43) After the waves of contagion, these challenges were gradually overcome. Failure to fully overcome these impacts can lead to a significant lack of coverage in certain geographic regions, as well as the overcrowding of surgical reference units.

### Study limitations and suggestions for future studies

Although the present study consisted of a significant sample of articles, it was not possible to exhaust all existing databases, academic search engines, portals and libraries, nor to carry out more than one crossing of descriptors. Furthermore, during the course of the study, some issues related to the challenges of current care practices in the context of surgical service units were identified. These issues pointed out and discussed may be objects of further research, such as strategies that have been proposed and discussed by scholars to overcome these challenges listed.

## Conclusion

The main challenges that surgical services have faced or continue to face in this period of the COVID-19 pandemic are the transfer of resources from operating rooms to other units and the hiring of professionals to meet the demands of health services that provide surgical care. Finally, discussing and reflecting on the challenges that have affected and continue to affect outpatient and hospital health services is important. Such reflections encourage professionals, who work on the front line and in the management of these services, to know the motivations that led surgical health services to adopt radical changes in a timely manner to offer safe care to patients affected by COVID-19.

The study started from the need to know the evident impacts of the COVID-19 pandemic on health sectors, especially in operating rooms. The results found and the discussions raised allow greater clarity about the challenges in surgical processes during the COVID-19 pandemic. In this regard, they can help health professionals, whether assistance or managers, in decision-making regarding the strategies that could and that can be traced in the face of the current scenario and the likely new waves of contagion of this disease.
